# Identification and Validation of Efficacy of Immunological Therapy for Lung Cancer From Histopathological Images Based on Deep Learning

**DOI:** 10.3389/fgene.2021.642981

**Published:** 2021-02-09

**Authors:** Yachao Yang, Jialiang Yang, Yuebin Liang, Bo Liao, Wen Zhu, Xiaofei Mo, Kaimei Huang

**Affiliations:** ^1^Key Laboratory of Computational Science and Application of Hainan Province, Haikou, China; ^2^Key Laboratory of Data Science and Intelligence Education (Hainan Normal University) Ministry of Education, Haikou, China; ^3^School of Mathematics and Statistics, Hainan Normal University, Haikou, China; ^4^Qingdao Geneis Institute of Big Data Mining and Precision Medicine, Qingdao, China; ^5^Geneis (Beijing) Co., Ltd., Beijing, China

**Keywords:** immunotherapy, lung cancer, convolutional neural network, biomarkers, DeepLRHE

## Abstract

Cancer immunotherapy, as a novel treatment against cancer metastasis and recurrence, has brought a significantly promising and effective therapy for cancer treatments. At present, programmed death 1 (PD-1) and programmed cell death-Ligand 1 (PD-L1) treatment for lung cancer is primarily recognized as an immune checkpoint inhibitor (ICI) to play an anti-tumor effect; however, it remains uncertain regarding of its efficacy though. Thereafter, tumor mutation burden (TMB) was recognized as a high-potential to be a predictive marker for the immune therapy, but it is invasive and costly. Therefore, discovering more immune-related biomarkers that have a guiding role in immunotherapy is a crucial step in the development of immunotherapy. In our study, we proposed a deep convolutional neural network (CNN)-based framework, DeepLRHE, which can efficiently analyze immunological stained pathological images of lung cancer tissues, as well as to identify and explore pathogenesis which can be used for immunological treatment in clinical field. In this study, we used 180 whole slice images (WSIs) of lung cancer downloaded from TCGA which was model training and validation. After two cross-validation used for this model, we compared with the area under the curve (AUC) of multiple mutant genes, TP53 had highest AUC, which reached 0.87, and EGFR, DNMT3A, PBRM1, STK11 also reached ranged from 0.71 to 0.84. The study results showed that the deep learning can used to assist health professionals for target-therapy as well as immunotherapies, therefore to improve the disease prognosis.

## Introduction

Lung cancer is currently one of the most common malignant tumors and the main cause of death in the world. The Global Cancer Statistics 2018: GLOBOCAN Estimates of Incidence and Mortality Worldwide for 36 Cancers in 185 Countries showed that the incidence and mortality of lung cancer in my country are 11.6 and 18.4%, respectively, and both ranked first in the world (Bray et al., 2018). By 2020, The American Cancer Society reported that approximately 228,820 lung cancer cases will be diagnosed in the United States and 135,720 people would die from the disease (American Cancer Society, 2020). Currently, surgical treatment such as lobotomy and chemotherapy still remain the first or optimal treatment plan for patients with lung cancer. However, those types of treatments are invasive and have intolerable complications which decrease the quality of life for patients. Therefore, it is very important for health professionals to develop a novel strategy to improve treatment outcomes and improve patient survival time.

In recent years, the emergence of immunotherapy has brought new hope for the cure of tumors. Immunotherapy can restore the balance of the immune system by blocking immune checkpoints, so that T cells can enhance or restore anti-tumor effects, therefore, patients do not have to destroy their own cells with normal cells together. The mechanism of immunotherapy is that cancer growth and spread are not only dependent on tumor cells alone, but also affected by the integrating with the body’s immune system. The immunotherapy for malignant tumor is to stimulate patients’ own immune system to recognize the specific membrane molecules or gene mutation of malignant cells with gene mutation, thereby to induce the tumor cell apoptosis and remove from the body. As immunotherapy has been introduced in clinical field for years, the 5 years survival rate of advanced lung cancer has been improved from less than 5 to 16%, significantly. Therefore, immunotherapy has been a potential candidate for the cancer treatment in the clinical field.

Immunotherapy of lung cancer has been previously failed to introduce on the clinical practice, since it is lack of sufficient load of mutated tumor antigen, suppressed antigen presenting cells (APC) traffic from the tumor, as well lack of the specific biomarker signal for delivering CD4 T cells. Tumor can escape by losing cell antigen or the antigen-presenting molecule MHC class I. PD-L1 is a membrane ligand in lung cancer which is expressed on tumor cells in approximately 50% of lung cancers, and its expression may contribute to poor prognosis by suppressing T-cell function and promoting tumor cell to escape from the body immune response. After binging to cells APCs presenting at tumor cells, the body activates immune response, by activin CD4 T helper cells, CD8 cytotoxic T, and to eliminate or apoptosis of tumor cells (Lesterhuis et al., 2011).

Recently, immunotherapy for lung cancer has sufficiently aroused people’s interest in checkpoint inhibitors, especially PD-1/PD-L1 immune checkpoint inhibitors (ICI). ICI works by regulating the integration of T cells and APC or tumor cells to help suppress the immune response. Compared with empirical therapy, it is more effective in treating patients with complications (Jonathan et al., 2019). To date, most immunological therapy is using antibody to PD/PD-L1, the efficacy depends on the type of tumor, side effect and clinical stage of tumors. FDA approved application PD-1/PD-L1 treatment in advanced squamous and non-squamous. In fact, the PD-1 checkpoint blockade is associated with smoking status, DNA repaired pathway and higher non-synonymous mutation burden, overall, it works better for any types of tumors in advanced stage (Zhang et al., 2018; Wang et al., 2019).

In the era of precision medicine, machine learning has promoted the rapid development of computer-aided diagnosis, and it significantly improved the accuracy and efficiency of doctors in diagnosing patients (Zou et al., 2018; Zou and Ma, 2020). In the lung cancer research, The School of Medicine at New York University used deep learning methods to train hematoxylin-eosin (H&E) slices of lung cancer, and then identified the biomarker genes using for the immunological therapy (Coudray et al., 2018). Immune biomarkers could provide valuable prediction and disease prognoses the process of immunotherapy (Gulley et al., 2017). In addition, Agajanian et al. (2019) used random forests, gradient boosted tree classifiers and deep convolutional neural networks to predict driver gene mutation in the genome data set. In the study, they conducted twice cross-validation, and the results further prove the ability of CNN to extract advanced features.

In order to better understanding the mechanism of the body response to the surrounding microenvironment, it is critical for early evaluation and development of surveillance program by using potential effective biomarkers. In this study, we proposed a novel immunotherapy model based on CNN to predict mutant genes for immunotherapy by analyzing histopathological images of lung cancer stained with H&E images. The predicted biomarkers would play an important role for clinical professionals with designing personalized treatment plans.

## Materials and Methods

### Data Preparation

In this study, we downloaded H&E tissue images of lung cancer from TCGA^1^. The downloaded H&E images were converted to the SVS format, professional pathologists identified the tumor region and boundary. They discarded the furry and blurred background, the unqualified images as well as the background containing many macrovesicles, inflammatory cells and micro-fibrils and other inferencing factors to ensure the relatively clear images for training (Wang D. et al., 2016).

The cBioportal website^2^ is an open platform for interactive exploration of multi-dimensional cancer genomics data, which greatly facilitates the processing and analysis of data by researchers (Cerami et al., 2012). We used cBioportal website to analyze immune-related biomarkers, including TP53, EGFR, STK11, polE, polD1, PBRM1, DNMT3A, and KRAS. Furthermore, we marked the relevant biomarkers of H&E images as 1, otherwise mark them as 0. We reviewed and counted the clinical data of patients in the International Cancer Genome Collaboration Group (ICGC).

### DeepLRHE Framework

**FIGURE 1:**
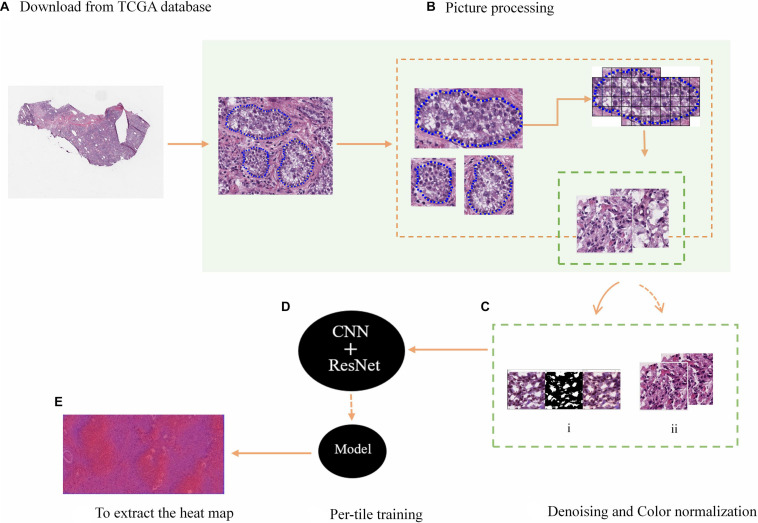
H&E tissue slices were processed and predicted immune-related biomarkers. **(A)** The WSI data from TCGA. **(B)** The selection and segmentation of the tumor area of images. **(C)** The denoising of the background influencing factors (i), the normalization of image color (ii). **(D)** The model training. **(E)** The heat maps.

In this study, the DeepLRHE framework was proposed on the basis of CNN, which can process H&E pathological slice images of lung cancer, as shown in Figure 1. It included four steps: annotate the tumor region in the image; standardize the color images; classify the samples; and identify the potential biomarkers in images.

The downloaded WSI was screened and the tumor area was depicted as shown in Figure 1B. Since the information contained in the complete tumor region is rich and complex, in order to facilitate training, a non-overlapping 512 × 512 window was used to segment images, while the small tiles containing tumor irrelevant features or unqualified images were discarded, the remaining were prepared for the subsequent processing. In addition, Python software and Gaussian mixture model was used to denoise and normalize the color of small tiles to ensure the quality of model training. The process is shown in Figure 1C. Then, input small blocks with immune-related biomarkers into the CNN+ResNet model with residual blocks for training and classification, as shown in Figure 1D. Finally, collect the results of all small tiles after induction and classification to extract a complete probability heat map.

### Image Preprocessing

We used H&E stained images of 180 WSIs with lung cancer downloaded from TCGA. H&E stained images were widely used for tumor diagnosis (Dalton et al., 2000; Le et al., 2012). Therefore, in order to be able to accurately predict the potential biomarkers on the images, an experienced pathologist would annotate the relatively accurate boundaries of the tumor region. The boundaries were shown as the blue dashed line in Figure 1B. In order to facilitate CNN training with images, a 512 × 512 window was used to scan and segment WSI, and small tiles with a background area greater than 75% are discarded.

### Denoising and Color Normalization

Background noise such as blank or flurry areas in the H&E slices may cause unclear image features and false positives results, which would significantly influence on model training. In order to address this problem, we used the OpenCV package in Python to remove image noise on H&E slices. We calculated the noise ratio threshold, which is the ratio of the area of the blank and blurred areas in the H&E images to the total area. According to this threshold, the false positive H&E slices were discarded, while retaining the images without background noises. OpenCV also performed edge expansion (filling), smoothing filtering and segmentation processing on H&E images, as shown in Figure 1C. Finally, all the processed image data is randomly divided into training set and validation set at a ratio of 1:1 to prepare for subsequent model training.

In addition, hematoxylin and eosin staining the nucleus and cytoplasm of the slices, might cause the color difference between each H&E slice, thereby to influence the model certain point during training (Bejnordi et al., 2016). In order to standardize the color of image slices without eliminating useful features, the Deep Convolutional Gaussian Mixture Model (DCGMM) was used to identify the color information of the nucleus, cytoplasm, and image background in the input H&E tissue image, and convert them into reference images the color (Zanjani et al., 2018). This method would not transform or change the original features in the image. The DCGMM model is a probability distribution model formed after the linear superposition of N-dimensional Gaussian mixture on the basis of the probabilistic Gaussian mixture model (GMM). Its specific form is as follows:

(1) $$P\,\left(x\right)=\sum_{n=1}^{N}W_{n}\,E\,\left(x|V_{n},\mathrm{Cn}\right)$$

Where the weight coefficient of the Gaussian mixture model of data x is *W*_*n*_, which needs to satisfy the condition $0\leq W_{n}\leq 1,\sum_{n=1}^{\text{N}}W_{n}=1$ (Bishop, 2006).

E is a normal distribution, mean V_n_ and covariance matrix C_n_ are its independent variables, then it satisfies the following formula for random variable x_K_:

(2) $$f\,\left(x_{k}\right)=\frac{1}{{\left(2\,\pi \right)}^{\frac{n}{2}}\,{\left|C_{n}\right|}^{\left(1/2\right)}}\,\exp\,\left\{\frac{-1}{2}\,{\left(x_{k}-V_{n}\right)}^{T}\,C_{n}^{-1}\,\left(x_{k}-V_{n}\right)\right\}$$

Where Cn-1 is the inverse matrix of C_n_, and the subsets of x_K_ are uncorrelated.

When W_n_ is *a priori* condition, the generation probability of the n -th model of x_K_ is:

(3) $$P\,\left(k,n\right)=\frac{W_{n}\,E\,\left(x|V_{n},C_{n}\right)}{\sum_{j=1}^{N}W_{j}\,E\,\left(x|V_{j},C_{j}\right)}$$

Input image data x, the total pixel value of sub-model x_k_(k = 1,2,3⋯) is E, according to formula (1), the (natural) log likelihood function can be obtained as:

(4) $$\mathrm{lnP}\,\left(x_{k}|W,V,C\right)=\sum_{k=1}^{k}\mathrm{lnP}\,\left(x\right)$$

Given CMM, the DCGMM model can be optimized by maximizing the log-likelihood function (formula 4) through the parameter (W_n_,V_n_,C_n_).

This color normalization method is chosen in unsupervised neural network. Unsupervised neural networks are the evolution of more detailed and tighter neural networks obtained from supervised learning. It pays more attention to the coordination between the internal units of the network. This shows that the use of the DCGMM model does not require any assumptions about the content of H&E images or data labels from the outside world, and the connection weight can be adjusted by itself (Sheng-chun and Lin-Xiang, 2006; Wei et al., 2016).

**FIGURE 2:**
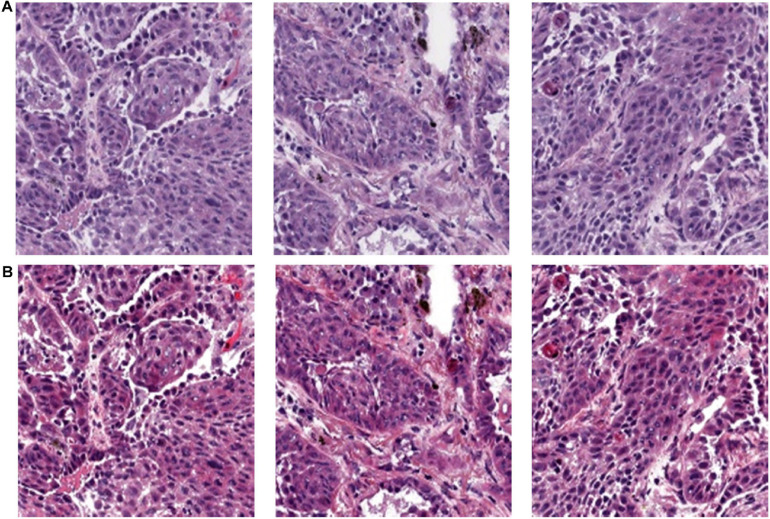
Color normalization. **(A)** H&E slice original images. **(B)** Color normalized images.

As shown in Figure 2, the original pictures of the H&E slice are in A. After applying DCGMM model, all the nuclei, cytoplasm, and patch backgrounds in picture A were classified into one type of pixel, so that images in B are obtained. This process would not discard the original feature of the pre-processed image.

### Convolutional Neural Network

**FIGURE 3:**
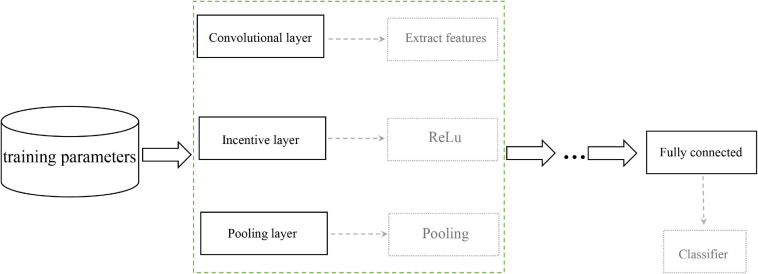
CNN training flowchart.

Convolutional Neural Network (CNN) is a multilayer neural network, which mainly includes input layer, convolutional layer, pooling layer and fully connected layer. It is considered to be the first choice for deep learning on the area of tumor diagnosis. The complete data processing was explained as followings: WSI enters the input layer, sequentially enters to the multiple convolution layers, and output from the merge layer, as shown in Figure 3. The activation function is commonly a RELU layer, and then followed by the pooling layer which is mainly to extract features for dimensional reduction through excitation layer (LeCun et al., 1998). The pathological or atypical features can be extracted and quantified through the CNN model. Furthermore, CNN could also discard the background noise and impurities from the pathological images, complete the segmentation and classification for the tumor region (Weinstein et al., 2009; Xu et al., 2014; Ertosun and Rubin, 2015). Therefore, CNN plays an irreplaceable role in processing pathological images, which is served as a convenient tool for the health professionals to make diagnosis and design individualized treatment (Song et al., 2015).

In order to accurately identify biomarkers of immunotherapy in lung cancer, the H&E images must be classified and trained. The preprocessed images with potential biomarkers were output as the input layer of the deep convolutional neural network, and then the feature extraction was performed through the convolutional layer composed of 32 n×n convolution kernels. In the excitation layer, we choose ReLU as the activation function, which could increase the sparsity of the network to solve the over-fitting problem, then the images passed through the pooling layer to extract features (Shang et al., 2016). The input data to the next hidden layer until the features in the image were completely extracted, and finally classified in the fully connected layer and as well as the output layer.

### Residual Network

Generally, neural networks can obtain better functions, but affected by the depth of the network layer. However, as the network continues to deepen, the convergence of the network would deteriorate due to the issue of vanishing gradient, and the accuracy and performance of the network will decrease. With improved convergence but not degraded network, we introduced the ResNet block.

**FIGURE 4:**
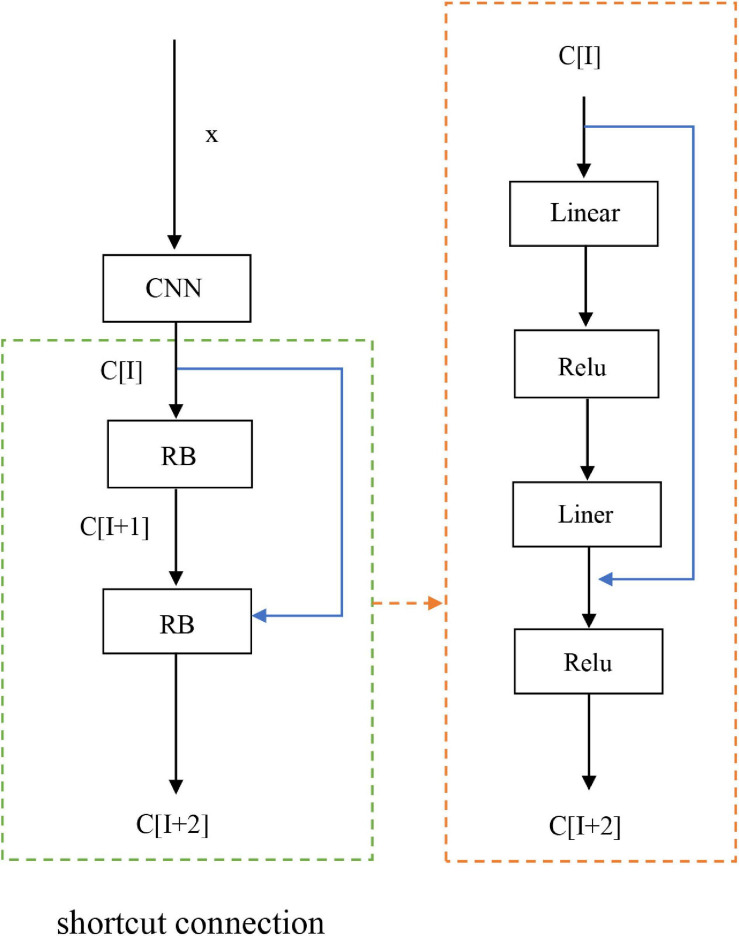
ResNet flowchart.

As a fairly deep network, ResNet has applied in image feature classification, lesion segmentation, cell segmentation etc. (Russakovsky et al., 2015; Shin et al., 2016; Wang C.W. et al., 2016). This method introduced ResNet on the basis of CNN. At this time, a fast connection was formed between various layers in the network, and they can accelerate the connection between different layers, as shown in Figure 4. The two layers are treated with shortcut connections as residual blocks (RB), and then the input vector of the considered layer is set to *x*, while the output vector is set to c. ResNet is equivalent to a special form of shortcut connection with the identity mapping. The activation function used in the network is the nonlinear function ReLu. The linear function is LC+A, from this we get C[1+2]= σ(B[W + 2] + C[1]) between layers, where B[W + 2] = W[1 + 2] * C[1 + 1] + A[1 + 1], when W[1 + 2] and A[1 + 1] = 0 are establishedit is easy to see that C[1 + 2] = σ(C[I]), when C[1] ≥ 0, C[1 + 2] = σ(C[1]) is true. At this point, the identity mapping was established. In addition, we added BN technique to residual network, because the BN can make the landscape of the entire loss function smoother, therefore to optimize the predictability and stability of the network. The addition of Resnet and BN techniques allowed our model to deepen the network level and training speed, while improving the classification accuracy, general ability, and expressive effect of the network (He et al., 2015; Shen et al., 2017; Santurkar et al., 2018).

### Adjustment of Hyperparameters

In order to optimize the performance of the model, we need to adjust the hyperparameters which had a variety of combinations. We choose to adjust the hyperparameters of the model under twofold cross-validation and take its optimal value. The data was further divided into a training set and a validation set. The model was trained with the training set, while the validation set was used to verify the performance of the model. Subsequently, we defined a grid of four dimensions and the possible range of each dimension. During the training process, the validation set and the network model would generate a mapping in time, and each mapping contained a hyperparameter, which indicated that the validation set can be adjusted by parameters of the model. These hyperparameters included the number of cores of the filter core, the number of layers, the batch size and the loss function of the convolutional neural network.

There were many types of hyperparameters and manual adjustment were required. Therefore, a simple test was required to determine the parameter adjustment range. Because the learning rate was difficult to be determined when the regularization term was introduced, the appropriate learning rate threshold was obtained afterward. The coefficient of regular term was recorded as 0, and a small number of samples can be obtained according to this threshold. We can obtain the approximate range of the hyper-parameters by adjusting the hyper-parameters in this step. In a wide range, we adjusted the learning rate and the regularization term coefficients to obtain the refinement, so as to obtain the optimal parameter values.

## Results

### Clinical Diagnosis Information

We downloaded 180 lung cancer WSIs from the TCGA database. We counted clinical variables associated with lung cancer incidence in Table 1, thereby to eliminate possible bias for influencing the biomarkers for immunotherapy. Among them, adenocarcinoma tends occur at older age, however, age of occurring of squamous cell carcinoma tends to no difference, the overall death is relatively younger, which indicated the lung cancer occurs in middle-aged and elderly people and the mortality rate is very high, especially in male.

**TABLE 1:** Clinical characteristics of the patients in this study.

Clinic-pathologic variables	Category	Variables
Gender	Male	99
	Female	81
Age at diagnosis	Mean	67
The types of the samples	H&E	100%
Adenocarcinoma	>67-year-old	59
	≤67-year-old	46
Squamous carcinoma	>67-year-old	42
	≤67-year-old	33
Age of death	Mean	68

### Data Preprocessing

**FIGURE 5:**
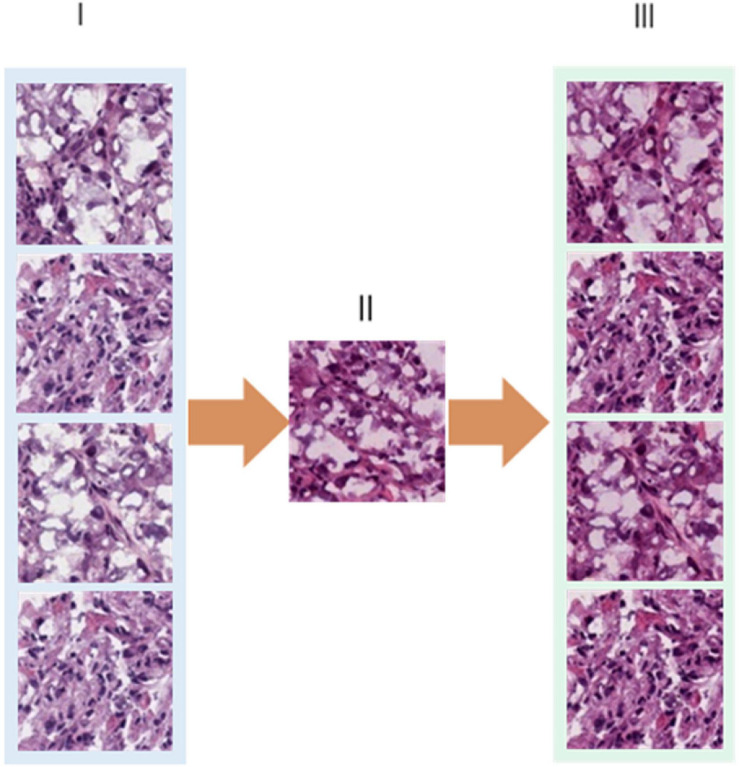
Color normalization model of tissue slice image. **(I)** Original slice image; **(II)** Reference image selected by professional pathologists. **(III)** Color standardized slice image after model processing.

The 180 images of lung cancer H&E sample data were downloaded from TCGA were all converted to SVS format, and the tumor area was annotated by a professional pathologist. In order to facilitate data training, each image was divided into small tiles of 512 × 512 size, as shown in Figure 1B. The blank areas and small areas with a background greater than 75% discarded, and small tiles with high image quality are selected for subsequent application, including imaging with uniform cytoplasm and nucleus staining, no background noise and less interference factors. We set the ratio of blank and blurred areas over the total area in the H&E image as a threshold, and used Open CV to process images with a threshold less than 75%. In the end, we left 1,800 qualified small tiles, and then used the DCGMM model to normalize the colors of these small tiles. The process and results were shown in Figure 5. After our image data was processed by the DCGMM model, the color was standardized, and the image features still remained in the original slice.

### Performance Evaluation

By using twofold cross-validation, we divide the pre-processed 1,800 samples into a test set and a validation set, and then the samples were classified and trained by the CNN + ResNet model. After training, the probability heat map of the H&E image with potential biomarkers in lung cancer immunotherapy were obtained, and then the average probability of each biomarker on the H&E image and their AUC value were obtained. Among them, TP53 obtained the highest AUC value, reaching 87%. The AUC value of EGFR also reached 84%, and the AUC values of DNMT3A, PBRM1, and STK11 were between 71 and 78%.

### Evaluation of Immunotherapy Biomarkers

**FIGURE 6:**
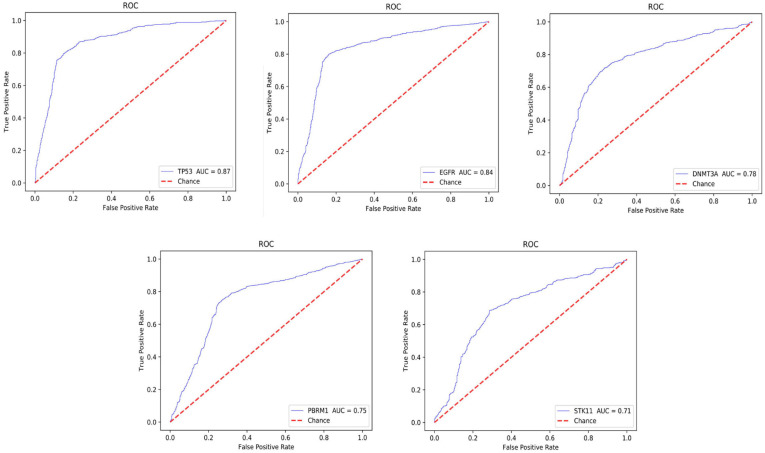
Gene ROC curve.

As shown in Figure 6, taking the false positive rate (FPR) as the X axis and the true positive rate (TPR) as the Y axis, the ROC curves of these 5 biomarkers are drawn based on the AUC value.

Our results have reached a very high accuracy. Among them, the AUC value of TP53 is 87%, which is the highest AUC value among our predicted biomarkers. TP53 mutations occur in about 50% of NSCLC, mainly targeting lung epithelial cells (Bodner et al., 1992). A large number of studies have shown that its mutation is closely related to the occurrence and treatment of lung cancer (Mogi and Kuwano, 2011). TP53 is an important tumor suppressor gene encoded the p53 protein, it could regulate cell proliferation, growth and repairing DNA damage. When TP53 gene is mutated, cell division and replication and proliferation, leading to tumor initiation.

The AUC value of epidermal growth factor receptor (EGFR) is slightly lower than that of TP53, which is 84%. It occurs mostly in female patients and non-smokers (Lai et al., 2013). EGFR is a large transmembrane glycoprotein, which can regulate the physiological processes of cell growth, proliferation and differentiation by combining with epidermal growth factor (EGF) in its extracellular domain. The overexpression, amplification and mutation activation of EGFR can induce cancer. In lung cancer, most of the mutations of EGFR are caused by the rearrangement and amplification of the EGFR gene or the selective splicing of mRNA (Cheng et al., 2012; Cheng and Eble, 2013).

The AUCs of DNMT3A, PBRM1, and STK11 are shown in Figure 6, which are 78.1%, 75.3%, and 71.6%, respectively. We described their physiological function as followings.

DNMT3A mutation is actually the main cause of blood system cancer, but recent studies have also proved its effect in lung cancer (Yuejing et al., 2014). DNA methyltransferase 3A (DNMT3A) is responsible for the methylation of human genes. DNMT3A gene mutation would result in the inactivation of tumor suppressor genes, and the damaged DNA cannot be restored in time, resulting in normal cell abnormalities. This process is closely related to the occurrence and development of tumors.

STK11 gene and the serine threonine kinase encoded by it are tumor suppressors, which regulate cell metabolism and growth through phosphorylation of adenosine monophosphate activated protein kinase (AMPK) and 12 AMPK-related kinases (Shackelford and Shaw, 2009). In animal studies of lung adenocarcinoma, it has been found that the inactivation or mutation of STK11 will be related to the change of the tumor microenvironment and the decrease of cytotoxic CD8^+^ cell infiltration, which will lead to the separation of the tumor from the body’s immune monitoring.

PBRM1 and STK11 are often regarded as negative markers because of their low expression rate, but in fact there is still a correlation between them and immunotherapy (Skoulidis et al., 2018).

### Heat Map Generation

**FIGURE 7:**
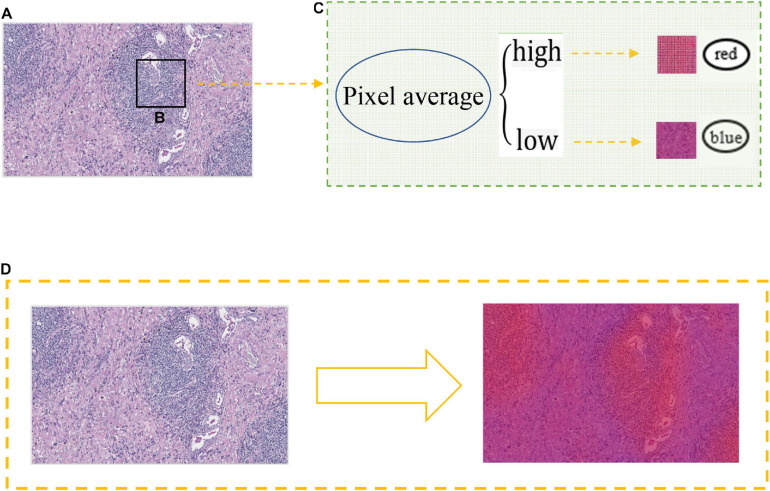
Heat map generation. **(A)** Probability image of H&E slice after training. **(B)** A 512 × 512 size non-overlapping window for scanning slice images. **(C)** The relationship between the average value of pixels and the color change of the heat map. **(D)** Generate a heat map.

In order to extract the heat map, we generated a probability map from the tumor region, then scanned the image with a 512 × 512 window, and obtained the result in this window through the CNN model, as shown in Figure 7. We applied the window result to the pixels and average the sum of all pixels in each window. High pixels indicated that the possibility of biomarkers on the image is high with the color in the heat map show as red, otherwise it was blue. Combined with AUC analysis, our model had a relatively good effect in predicting immune-related biomarkers by using H&E lung cancer slices.

## Discussion

With the continuous development of artificial intelligence in the medical field, deep learning as an important method has been able to effectively identify and analyze disease images. In our study, five immune biomarkers TP53, EGFR, DNMT3A, PBRM1, and STK11 were identified from the H&E slice images of lung cancer. The AUC value is shown in Figure 6. Patients with TP53 mutation relatively not response to target therapy. Studies have confirmed that when patients with non-small cell lung adenocarcinoma have TP53 gene mutations, also accompanied by an increased PD-L1 expression, which often indicates those patients are more likely sensitive to PD-1/PD-L1 therapy (Dong et al., 2017). From the perspective of the biological mechanism, TP53 deletion can cause an increase immunogenicity of tumor, further increasing in cytotoxicity of T lymphocytes (CD8^+^ T cell). Several recent clinical trials found that TP53 mutation can predict (PFS) of progression-free survival (Hellmann et al., 2018; Assoun et al., 2019). Combining those biological mechanism, our studies confirmed that TP53 could be a potential biomarker with for lung cancer immunotherapy.

EGFR gene mutations are mostly caused by the deletion of L858R and exogenous factor 19, which will change the specific mutations of EGFR protein. It has been confirmed that patients with EGFR mutant primarily benefit with from targeted therapy with tyrosine kinase inhibitors (TKI) (Recondo et al., 2018; Yang et al., 2019). Studies have showed that the use of ICI as an adjuvant therapy in patients with drug-resistant, non-small cell lung cancer after EGFR-TKI treatment, it can make the patients increase patients’ survivals (Wu et al., 2017; Garassino et al., 2020). Moreover, when EGFR21 exon combined with L858R mutation and EGFR20 exon were treated with ICI, they all response rate (International Association for the Study of Lung Cancer, 2017). Therefore, this provides the possibility for the application of immunotherapy in some EGFR mutations.

Mutations in the DNMT3A gene will result in the inactivation of tumor suppressor genes, dysfunction in repairing DNA, resulting in abnormal function of cell. DNMT3A has been proven to be a risk factor for rectal cancer, blood cancer and ovarian cancer etc. A recent study on the side effects of immunotherapy found that EGFR and DNMT3A are associated with Hyper-progressors (Champiat et al., 2017; Kato et al., 2017). The reason may be inactivation of Janus kinase 1 (JAK1), which leads to the decrease of PD-L1 expression, thereby tumor cells lack the sensitivity to PD-1 antibody treatment, resulting in inherent drug resistance. It may also be β-2 It is caused by a truncated mutation in the gene encoding macroglobulin (B2M), but this reason is just a guess, and the real reason has yet to be confirmed.

STK11 is the main driving gene for the primary resistance of PD-1 inhibitors, and its deletion will promote the resistance of PD-1/PD-L1 inhibitors (Papillon-Cavanagh et al., 2020). The results also highly indicate the possibility of STK11 gene mutation as a prognostic indicator of anti-PD-1/PD-L1 therapy in lung adenocarcinoma.

PBRM1 is a promising biomarker for kidney cancer. When CcRCC was treated by ICI, found that patients with PBRM1 mutation are more sensitive to PD-1 antibody (Braun et al., 2019). However, lung cancer patients with mutant PBRM1 benefit less from treatment-related survival. As shown in Figure 6, although the AUC level is not high, it still has the potential to become a negative biomarker.

Immunotherapy is a relatively new concept in cancer treatment, since it advantages in less tolerated, high efficacy, it has quickly become a research hotspot. At present, there are no precise biomarkers for immunotherapy that can exert efficacy, and the role of each biomarker in the process of immunotherapy still needs clinical research. According to reports, numbers of clinical trial studies have shown that the use of immunotherapy in combination therapy has more advantages (Blumenthal et al., (2018); Jotte et al., 2020).

This study, we conduct deep learning CNN model, the model performance is relatively good by two-cross-validation. Therefore, it has important significance of immunotherapy in clinical practice. However, our study still has some limitation, there are still some images that contain features that are not easily recognized by the training model, which makes it difficult to classify, more due to the limited training data and the diversity of lung carcinogenic factors, there are some mutation samples in certain genes cause data unbalance. Moreover, we did not set an independent set to verify the model, these factors have a certain impact on the accuracy of our results.

## Data Availability Statement

Publicly available datasets were analyzed in this study. This data can be found here: the TCGA-LUSC and TCGA-LUAD projects in TCGA (https://portal.gdc.cancer.gov/) provide data to support the conclusions of this article. The diagnosis information of the patients in this article comes from the information on lung cancer of Americans in ICGC. Address For https://dcc.icgc.org/releases/release_28/Projects.

## Author Contributions

JY, BL, and WZ conceived the concept of the work. YY, KH, and YL collected or analyzed the data and performed the literature search and designed the experiments. YY, XM, and JY wrote the manuscript. All authors approved the final manuscript.

## Conflict of Interest

JY, YL, and XM were employed by company Geneis (Beijing) Co., Ltd. The remaining authors declare that the research was conducted in the absence of any commercial or financial relationships that could be construed as a potential conflict of interest.

## Footnotes

1 https://portal.gdc.cancer.gov/

2 http://www.cbioportal.org/
